# Formation, Microstructure, and Conductivity of a Novel Ga_2_S_3_-Sb_2_S_3_-AgI Chalcogenide System

**DOI:** 10.1038/s41598-018-20144-3

**Published:** 2018-01-26

**Authors:** Xinyu Huang, Qing Jiao, Changgui Lin, Hongli Ma, Xianghua Zhang, Erwei Zhu, Xueyun Liu, Shixun Dai, Tiefeng Xu

**Affiliations:** 1 Key Laboratory of Photoelectric Detection Materials and Devices of Zhejiang Province, Ningbo University, Ningbo, 315211 China; 2 Laboratory of Glasses and Ceramics, Institute of Chemical Science UMR CNRS 6226, University of Rennes 1, Rennes, 35042 France

## Abstract

Novel glasses in a Ga_2_S_3_-Sb_2_S_3_-AgI system were prepared with a melt-quenching method, and their glass-forming region was identified. The maximum dissolvable AgI in glasses was 65 mol%. The thermal, optical, and structural properties of glasses were investigated as a function of AgI and Ga_2_S_3_ contents. The Ga_2_S_3_-Sb_2_S_3_-AgI glasses possess a wide region of transmission window (0.65−14 μm). An ionic conductivity of approximately 1.01 × 10^−3^ S/cm can be obtained for a 40 (0.8Sb_2_S_3_-0.2Ga_2_S_3_)-60AgI glass at an ambient temperature, and the ionic conductivity increased as temperature increased. The relative activation energy of Ag^+^ conduction was also calculated. These novel glasses show potential for the combined application of infrared optics and solid electrolytes.

## Introduction

The development of large-scale energy systems has been widely explored because of the rapidly increasing demand for portable electronic devices, electric vehicles, and systems based on renewable energy. However, the use of large-scale batteries has raised safety issues associated with flammable organic liquid electrolytes and leakage hazards^1,2^. Thus, much effort has been devoted to creating new materials for all-solid-state rechargeable batteries^3–8^. Inorganic structures that provide suitable pathways for fast ion migration are possible solid electrolytes in next-generation solid-state energy conversion/storage, switching, and sensing devices^9–11^. Compared with glassy electrolytes, commonly used organic polymers or liquid electrolytes are often limited by practical problems, including relatively poor thermal and chemical stability, flammability, and relatively high operational voltages^3,9^. Glass electrolytes can be easily fabricated into complex shapes and possess a wide composition flexibility to optimize their properties and conductivities. Therefore, they are feasible electrolyte materials in solid-state batteries.

Despite the advantages of glass electrolytes, low ionic conductivities and high interfacial resistance are key issues that should be solved. Sulfide solid electrolytes have been extensively investigated not only because of the discovery of considerably high ionic conductivities (>10^−4^ S/cm) at ambient temperature but also because of low grain-boundary resistance^3^. Among various chalcogenide glass systems, various silver-containing chalcogenide glasses show high ionic conductivities of 3.2 × 10^−4^ S/cm in 40Ag_2_Se-10Ga_2_Se_3_-50GeSe_2_^12^ and 6.37 × 10^−4^ S/cm in 55(0.6GeS_2_-0.4Sb_2_S_3_)-45AgI^13^ at room temperature. The ionic conductivity of chalcogenide glasses can be enhanced by several orders of magnitude by increasing the concentration of AgI with a relatively small cation and plolarizable anion^14^.

The ionic conductivity of chalcogenide glasses is mainly reported for sulfur-based glasses because the mechanical properties of sulfide glasses are commonly better than those of selenide or telluride glasses due to the stronger bond between constituent atoms of the former than that of the latter. Therefore, sulfide glasses are favorable for practical applications. A Ga-Sb-S glass is commonly known for its transmission range in sulfur-based glasses. A large amount of CsI can be introduced to a Ga-Sb-S chalcogenide matrix and thus improve its glass-forming ability^15^. Adding AgI to sulfide glasses can also enhance their glass-forming ability and non-linear properties^16^. Therefore, to improve the glass-forming ability and to find new optic materials and chalcogenide solid electrolytes with high Ag^+^ ionic conductivity, we investigated a series of Ga_2_S_3_-Sb_2_S_3_ glasses, which exhibit good glass-forming ability and properties^17,18^.

In this study, the incorporation of silver iodine into a Ga-Sb-S glass was investigated, and the glass-forming region was determined. The thermal and optical properties of the formed glasses were reported. The ionic conductivity and structure of the glasses were examined through impedance and Raman spectroscopies, respectively. An expected ionic conductivity of 1.01 × 10^−3^ S/cm at room temperature (30 °C) was observed. To the best of our knowledge, this value is optimal for sulfur-based glasses.

## Results and Discussion

### Glass-Forming Region

**Figure 1:**
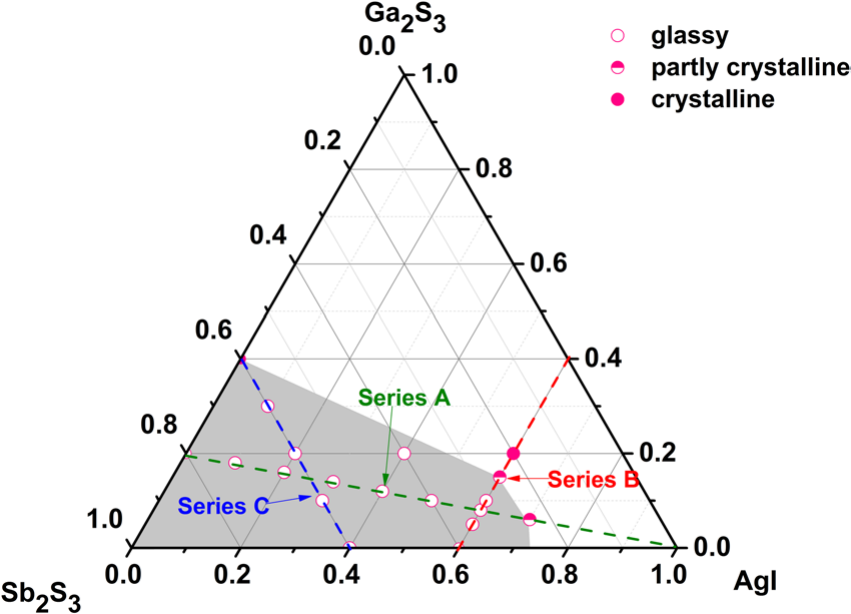
Glass-forming region of the Ga_2_S_3_-Sb_2_S_3_-AgI system. Series A: (100–*x*) (0.8Sb_2_S_3_-0.2Ga_2_S_3_)-*x*AgI, *x* = 10, 20, 30, 40, 50, 60, 65 and 70; Series B:(40–*y*)Sb_2_S_3_-*y*Ga_2_S_3_-60AgI, *y* = 5, 10, 15, 20; Serious C: 60Sb_2_S_3_-*z*Ga_2_S_3_-(40–*z*)AgI, *z* = 10, 20, 30.

**Figure 2:**
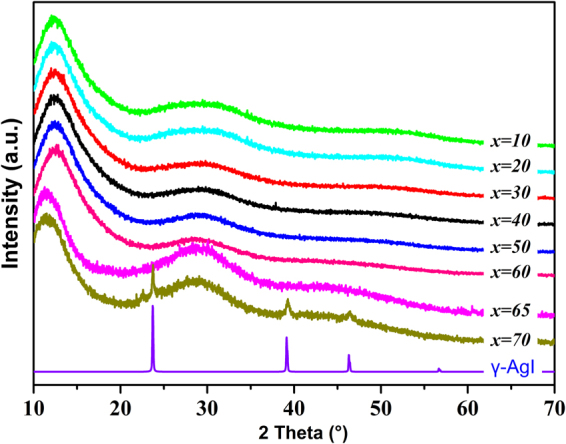
XRD patterns of glass samples in Series A (100 – *x*) (0.2Ga_2_S_3_-0.8Sb_2_S_3_)−*x*AgI: where *x* = 10, 20, 30, 40, 50, 60, 65, and 70.

Figure 1 shows the glass-forming region of the Ga_2_S_3_-Sb_2_S_3_-AgI pseudo-ternary system, which was determined on the basis of visual and XRD measurement. The obtained glasses are black, opaque, and homogeneous. The following series is observed: series A based on (100 − *x*) (0.8Sb_2_S_3_-0.2Ga_2_S_3_)- *x*AgI compositions with *x* = 0, 10, 20, 30, 40, 50, 60, 65, 70; series B based on (40-*y*)Sb_2_S_3_-*y*Ga_2_S_3_-60AgI with *y* = 0, 5, 10, 15, 20; and series C based on 60Sb_2_S_3_–*z*Ga_2_S_3_ - (40 − *z*)AgI with *z* = 0, 10, 20, 30. Figure 2 displays several XRD patterns of glass samples in series A. The amount of dissolved AgI can reach 65 mol% when the ratio of Sb_2_S_3_ to Ga_2_S_3_ is 4/1 (series A), and the crystallized phase γ-AgI is precipitated in a AgI-rich region. In this case, a high amount of AgI possibly facilitates an increase in ionic conductivity, as described in the next section. The glass system contains a large glass-forming region, which is larger than that of the Ga-Sb-S glass matrix^19^. This region is mainly situated in an Sb_2_S_3_-rich area and extended to the AgI-rich region. A large glass-forming region provides a wide compositional flexibility to optimize properties and conductivities. A previous research on similar chalcogenide glass systems^20,21^ indicated that the introduction of AgI to Ga_2_S_3_-Sb_2_S_3_ can improve glass-forming ability and ionic conductivity because of the formation of [GaS_4−x_I_x_] structure units and a large amount of Ag^+^, respectively. For series B, when the AgI content is fixed at 60 mol%, with respect to the Sb_2_S_3_-Ga_2_S_3_ component, the ratio of Sb/Ga is not flexible, and the glass likely crystallizes at a high Ga content.

### Thermal and physical properties

The thermal characteristics and some physical properties of the samples are given in Table 1. The glass transition temperature (*T*_g_) of the studied glasses range from the AgI transition (β/γ phase to α phase) temperature of 147 °C to 237 °C. In general, *T*_g_ is associated with the connectivity or degree of cross-linking of a glass network^22^. For the samples in series A, *T*_g_ decreases with *x*. Iodine atoms play a non-bridging role and decrease glass network connectivity^13^. This result is consistent with the decrease in network connectivity when AgI addition ranges from *x* = 10 to *x* = 65. Conversely, Δ*T*, or the difference between *T*_x_ and *T*_g_, is known as a critical parameter used to evaluate the thermal stability and fiber-drawing ability of a glass. Δ*T* in series A increases with *x*, indicating that introducing iodide to the Ga_2_S_3_-Sb_2_S_3_ glass progressively breaks Ga-S bonds, thereby forming a [GaS_4−x_I_x_] tetrahedral; the formation of this tetrahedral complex possibly favors glass formation^23^. For the compositions in series B, *T*_g_ decreases as the Ga_2_S_3_ content increases when the AgI content is fixed at 60 mol%. This phenomenon may be influenced by high Ga contents. As the amount of AgI in series A increases, density increases and hardness decreases. The evolution of density is easily understood because of the heavy mass of AgI, and the variation of hardness is due to the addition of iodine atoms. As discussed in a previous section, iodine atoms act as a glass network terminator and widely open the network, resulting in a decrease in hardness.

**Table 1:** Thermal and Physical properties of Ga2S3-Sb2S3-AgI Glasses.

Compositions	*T*_*g*_ (*°C)*	*T*_*x*_ (°*C)*	*ΔT (*°*C)*	*ρ (g/cm*^3^)	*Hv (kg/mm*^2^)
***Serious A: (100*–*x)(0.8Sb***_**2**_***S***_**3**_**-*0****.***2*Ga***_***2***_***S***_**3**_**)**-***xAgI***					
*x* = 10	227.7	348.2	120.5	4.234	198
*x* = 20	215.5	341.4	125.9	4.288	196
*x* = 30	202.8	336.5	133.7	4.453	186
*x* = 40	187.6	323.3	135.7	4.599	180
*x* = 50	171.5	300.7	129.2	4.810	175
*x* = 60	155.5	297.5	142.0	4.982	173
*x* = 65	149.7	287.6	137.9	5.086	168
***Serious B: (40***-***y)Sb***_**2**_***S***_**3**_**-*yGa***_**2**_***S***_**3**_**-*60AgI***					
*y* = 5	156.0	288.8	128.8	4.985	196
*y* = 10	151.0	279.6	128.2	4.936	190
*y* = 15	147.4	273.3	125.5	4.874	186
***Serious C: 60Sb***_**2**_ ***S***_**3**_ **-** ***zGa***_**2**_ ***S***_**3**_ **-** ***(40*** **-** ***z)AgI***					
*z* = 10	204.8	308.9	104.1	4.480	170
*z* = 20	218.6	312.5	93.9	4.271	151
*z* = 30	236.7	305.6	68.9	4.060	135

### Optical properties

**Figure 3:**
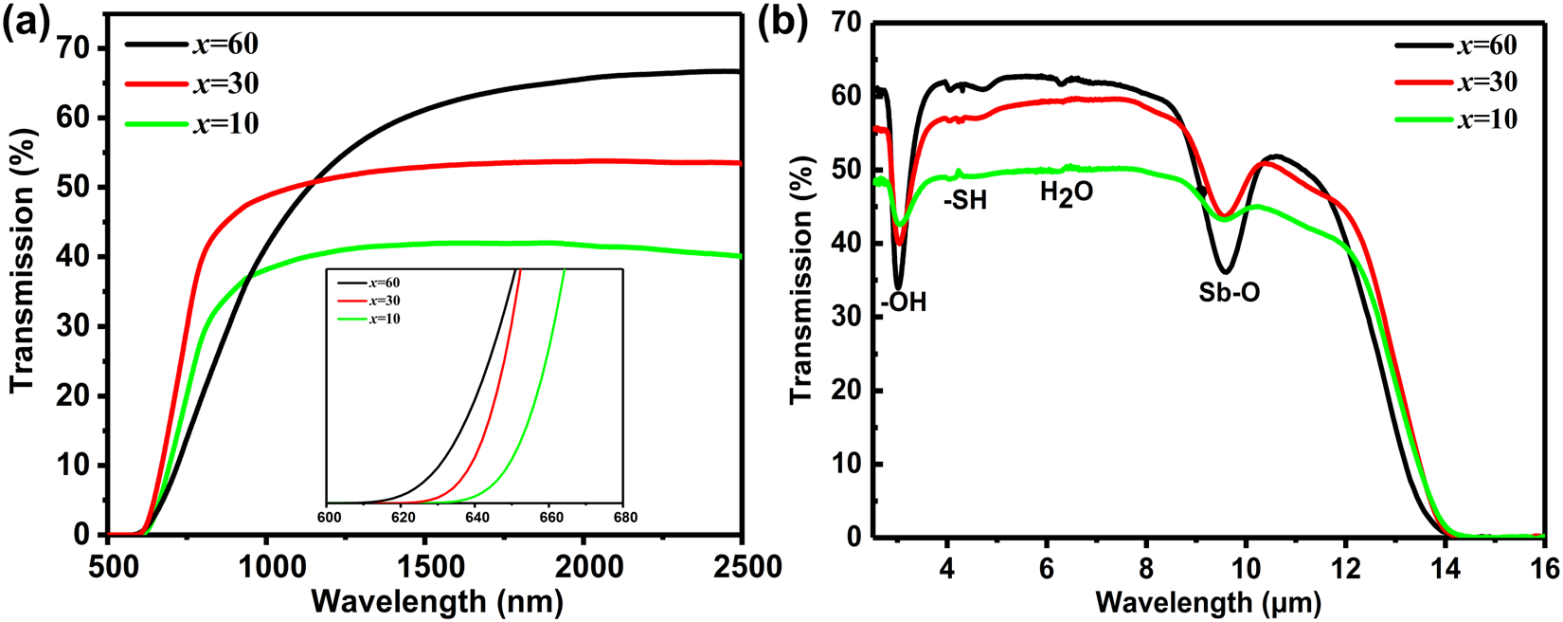
(**a**) Vis/NIR and (**b**) IR transmission spectra on Series A (100–*x*)(0.2Ga_2_S_3_-0.8Sb_2_S_3_)-*x*AgI: where *x* = 10, 30, and 60, respectively.

Figure 3 shows the vis-IR transmission spectra on series A: (100−*x*) (0.8Sb_2_S_3_-0.2Ga_2_S_3_)-*x*AgI. The plot includes the spectra for the samples containing 10, 30, and 60 mol% AgI. These glasses yield good transparency to approximately 14 μm, which is much longer than that of Ge-based chalcogenide glasses. Short wave absorption edge (λ_vis_) is attributed to the electrical transition between a valence band and a conduction band. In Fig. 3(a), λ_vis_ exhibits a blue shift as the AgI content increases. This phenomenon can be attributed to the addition of I^−^ with a lower polarizability (7.1 × 10^−24^ cm^3^) than that of S^2−^ (10.1 × 10^−24^ cm^3^)^24^. Such low polarizability of I^−^ addition results in an increase in the band gap between the valence band and the conduction band, and this phenomenon corresponds to a blue shift of λ_vis_. In Fig. 3(b), the absorption bands at 2.9, 4.1, 6.3, and 9.6 μm can be attributed to the existence of -OH, -SH, H_2_O, and Sb-O bonds, respectively^25^. These IR impurity absorption bands are enhanced as the AgI content increases because of the hygroscopic property of AgI. And it appears the glass with higher AgI shown decrease in transparecy at longer wavelength than other two glass. This might associated with the phonon energy of Sb-O is lager than that of Sb-S. Hence, purification technology^26,27^ should be applied to eliminate impurities in optics. In addition, the transmittance of glasses would increase with the addition of AgI, which is in accordance with phenomenon in Ge-Ga-S-AgCl system^28^.

### Glass structure

**Figure 4:**
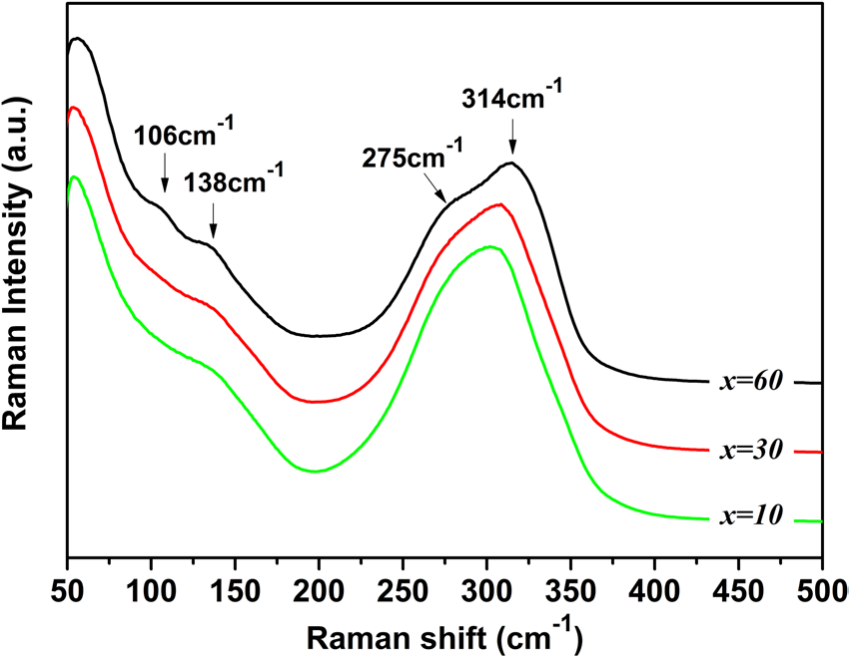
Raman spectra of glass samples in Series A (100–*x*) (0.2Ga_2_S_3_-0.8Sb_2_S_3_)−*x*AgI: where *x* = 10, 30, and 60, respectively.

Figure 4 illustrates the Raman spectra of three samples with 10, 30, and 60 mol% AgI on series A to elucidate the structural evolution of the studied glasses and develop a clear relationship between the chalcogenide glass structure and the ionic conductivity. The Raman spectra are dominated by a band between 50 and 350 cm^−1^, which are composed of several overlapping bands. According to previous research on 80Sb_2_S_3_-20Ga_2_S_3_ glass, the broad overlapping peak at about 300 cm^−1^ is assigned to the [SbS_3_] pyramid (290 and 314 cm^−1^), the shoulder around 138, 342, and 265 cm^−1^ to the [GaS_4_] tetrahedral structure units, and the [S_3_Ga-GaS_3_] ethane-like unit.^17,29^ Gallium tends to be coordinated with sulfur in chalcogenide glasses in fourfold; consequently, [S_3_Ga-GaS_3_] ethane-like units form to compensate for the shortage of sulfur in the 80Sb_2_S_3_-20Ga_2_S_3_ glass^9^. Metal halides, such as AgI, function as a glassy network terminator. When AgI is added to the 80Sb_2_S_3_-20Ga_2_S_3_ glass, the Ga-Ga bond in the ethane-like subunits of [S_3_Ga-GaS_3_] is gradually converted into a [GaS_4−x_I_x_] mixed structure because the Ga-I bond preferably forms rather than the Ga-S bond. With [GaS_4−x_I_x_] tetrahedral structure units, the glass framework becomes considerably open and provides a loosened glassy network environment for the diffusion of Ag ions. For the 40 (0.8Sb_2_S_3_-0.2Ga_2_S_3_)-60AgI glass, two new distinct peaks at 106 and 275 cm^−1^ are observed in the spectra. The appearance of the peak at 275 cm^−1^ is ascribed to the formation of the [SbS_3_-_x_I_x_] mixed structure according to vibration theory^30^. The new band at 106 cm^−1^ is attributed to γ-AgI at room temperature as the AgI concentration increases^31^. These structures may provide a basis for the understanding of the electrical conductivity of existing glasses.

### Conductivity properties

**Figure 5:**
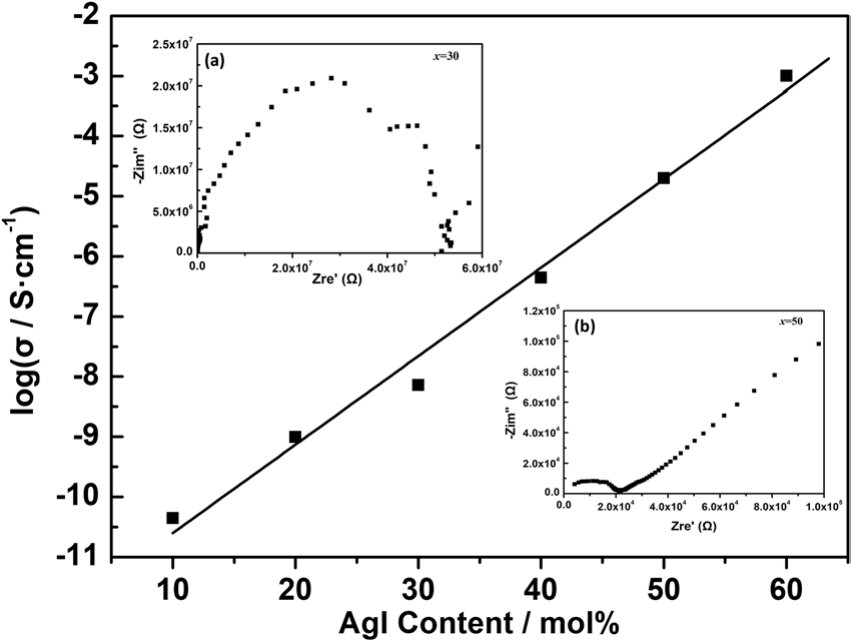
Ionic conductivity at room temperature (30 °C) of the glassy samples in Series A as a function of AgI content. The inset (**a**) and (**b**) present the Nyquist plots of impedance measured for the glass with composition of 70(0.2Ga_2_S_3_-0.8Sb_2_S_3_)−30AgI and 50(0.2Ga_2_S_3_-0.8Sb_2_S_3_) −50AgI, respectively.

**Figure 6:**
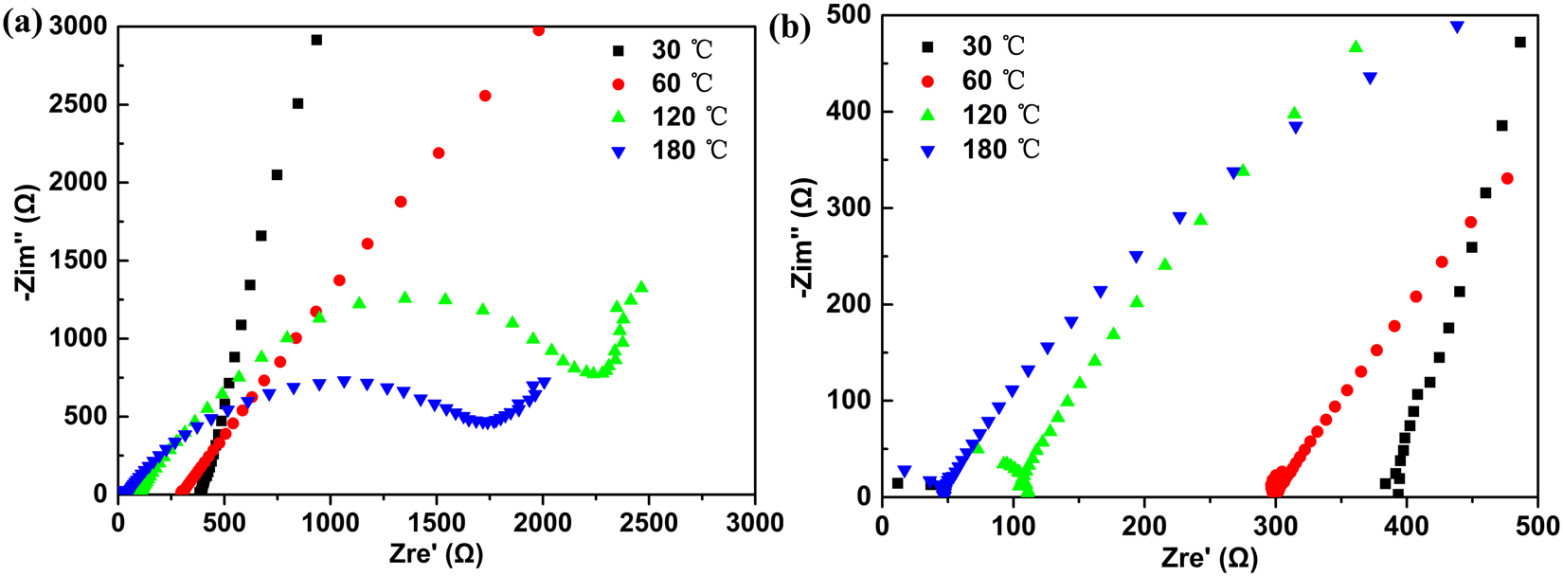
(**a**) Representative Nyquist plots of 40 (0.2Ga_2_S_3_-0.8Sb_2_S_3_)−60AgI glass at 30 °C, 60 °C, 120 °C, and 180 °C. (**b**) The magnified image of Nyquist plots of 40(0.2Ga_2_S_3_-0.8Sb_2_S_3_)−60AgI glass at high frequency region.

Figure 5 presents the compositional dependence of ionic conductivity at 30 °C for the glasses of series A. By increasing the Ag-ion concentration, the conductivity of glasses in series A monotonously increases, a.c. resistance can be calculated from the typical Nyquist plots of complex impedance as shown in the inset (a) and (b) of Fig. 5. As mentioned above, large amounts of AgI may provide superionic glasses. According to a study on a (1 − *x*)Sb_2_S_3_-*x*AgI system, within low temperature one the maximum of ionic conductivity appear at a AgI mole fraction of 0.6^32^. Thus, the conductivity of the 40(0.8Sb_2_S_3_-0.2Ga_2_S_3_)-60AgI compositions at 30 °C–180 °C is examined. Figure 6(a) displays the complex impedance spectrum of the examined glass, that is, the representative Nyquist plots at 30 °C, 60 °C, 120 °C, and 180 °C. Figure 6(b) presents a magnified image of the high-frequency region in Fig. 6(a). In the spectrum, Zre′ and −Zim″ are the real and imaginary components of the impedance Z(ω), respectively. The impedance spectrum of all of the samples usually shows three distinct features^33^. The first semicircle at a high frequency, which can be observed in Fig. 5(b), corresponds to the ionic conductive paths in a glass bulk, and the second semicircle denotes the double layer of electrode/electrolyte and the resistance of sampler holder and cables. The third segment in the low-frequency region follows a Warburg-type impedance, which is associated with a capacitive behavior similar to blocking electrodes (linear spike in the low-frequency region), indicating a typical ionic conductor. In theory, the total resistive impedance of bulk electrolytes can be deduced from the cross point of an arc with a horizontal line^3^. The diameter of the first arc decreases as temperature increases, and the intersection point moves to a low Zre′. This finding suggests that the resistance of the bulk glass decreases with temperature and conductivity increases. These phenomena are likely attributed to two factors. First, the increasing temperature contributes to the conduction of Ag^+^. Second, impedance plots are measured from a low temperature to a high temperature. Changes in the heat-treating induced microstructure also promote ion transport^33^. The resistance of the 40(0.8Sb_2_S_3_-0.2Ga_2_S_3_)-60AgI bulk glass obtained from the local minimum at the intersection of the plot is as low as 395 Ω.

Glass conductivity can be calculated using σ = *L*/(*SZ*_0_), where *L* is the thickness of electrolytes and *S* is the electrolyte-electrode surface area^16^. We assign the intercept on the real component of the impedance curve plot as *Z*_0_. The conductivity (σ) calculated from the total resistance is σ = 1.01 × 10^−3^ S/cm at room temperature (30 °C), which is one magnitude higher than that of 40GeSe_2_-30Ga_2_Se_3_-60AgI glass^16^. To the best of our knowledge, this value is one of the optimum results among Ag^+^ conductors for sulfides, which can be satisfied with the criterion of a fast ion conductor. Based on these experimental results and analyses, our conclusion is that the relatively high ionic conductivity in the study can be attributed to mobile Ag ions in glasses. On the one hand, the conductivity of the chalcogenide glass increases as the concentration of Ag ion increases. The maximum dissolvable silver iodine in this glass system is 65 mol%, which provides a large number of mobile Ag^+^. Otherwise, the addition of I^−^ with high electronegativity forms [GaS_4−x_I_x_] tetrahedral structure units. Consequently, the glass framework becomes open and provides a loosened glassy network environment, which also helps large amounts of Ag^+^ conduct through the glass matrix at room temperature.

**Figure 7:**
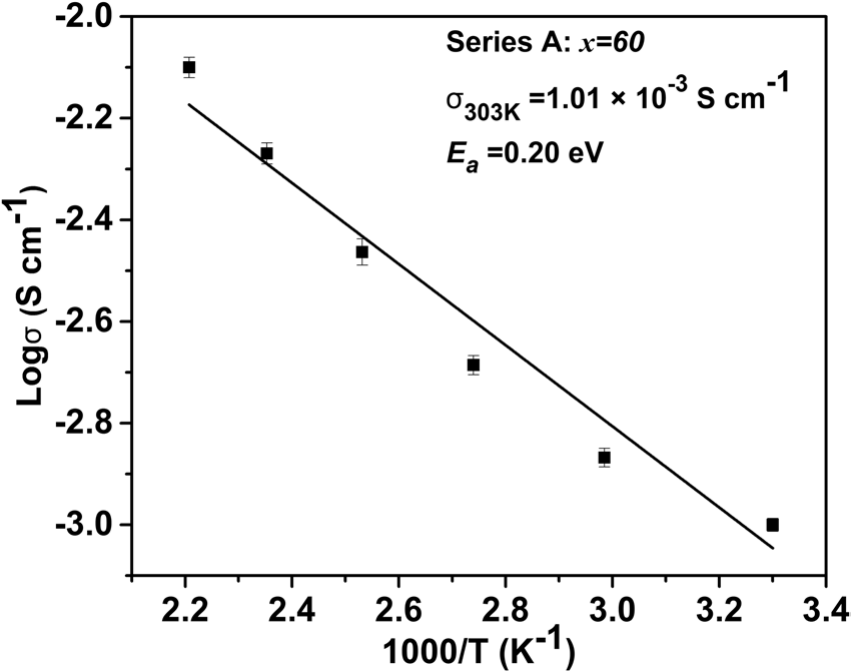
Temperature dependence of the conductivity of 40(0.2Ga_2_S_3_-0.8Sb_2_S_3_)−60AgI glass.

Figure 7 shows the reciprocal temperature dependence of the glass sample of ionic conductivity. The linear dependence of logσ versus (1/*T*) follows Arrhenius law and indicates phase stability at a given temperature. The activation energy (*E*_*a*_) of Ag^+^ conduction can be determined from the slope of the linear Arrhenius plot by using the following equation:

$$\sigma =Aexp(-{E}_{a}/{k}_{b}T)$$

where *A* is the pre-exponential parameter and *k*_*b*_ is the Boltzmann constant. The conductivity of the glass increases according to Arrhenius formula, suggesting that conductivity can be described by single mechanism. The calculated *E*_*a*_ of 40 (0.8Sb_2_S_3_-0.2Ga_2_S_3_)-60AgI glass is obtained from the linear fit of the data to Eq. (1) (Fig. 6). For the studied glass, the corresponding *E*_*a*_ is 0.20 eV, which is lower than that of the widely examined glass Na_3_PSe_4_^3^ which is 0.21 eV. Further experiments will focus on improving the mobility of Ag^+^ by designing various compositions to obtain optimal diffusion channels.

In summary, Ga_2_S_3_-Sb_2_S_3_-AgI chalcogenide glasses are investigated for optics and solid electrolyte applications. The glass-forming region of this system is determined, and 65 mol% AgI can be dissolved in this glass system. These glasses yield an excellent transmission from 650 nm to 14 μm, which make them good materials for IR optics applications. The high Ag^+^ conductivity of 1.01 × 10^−3^ S/cm at room temperature can be obtained for the 40 (0.8Sb_2_S_3_-0.2Ga_2_S_3_)-60AgI sample. This result indicates that this glass system shows potential for amorphous solid electrolyte applications.

## Methods

### Glass preparation

Chalcogenide glasses were prepared by using a classical melting mixture of highly pure raw materials (Ga, Sb and S of 5 N, and AgI of 4 N) in a sealed silica ampoule vacuum (~10^−3^ Pa). A sealed ampoule with an inner diameter of 10 mm was placed in a rocking furnace. The raw materials were heated from 25 °C to 850 °C at a 2 °C/min heating rate, maintained at this temperature for 12 h in the rocking furnace, and further equilibrated at 700 °C. To remove the inner constraints during rapid quenching, we swiftly transferred the alloys to a preheated furnace and annealed them at *T*_g_ of −30 °C for 5 h. The bulk glass was obtained by removing it from the ampoule, and the glass rod was finally cut and polished into disks of 10 mm in diameter and 2 mm in thickness.

### Characterization of samples

The amorphous characteristics of the samples were confirmed through X-ray diffraction (Bruker D2 phaser, λ = 0.15406 nm, 30 kV, 10 mA, Cu*Κ*α) in a reflection mode at room temperature in the 2θ range of 10°–70°. Glass characteristic temperature, including *T*_g_ and onset temperature of crystallization (*T*_x_), were identified by heating 10 mg of the sample in a hermetic aluminum pan at a 10 °C/min rate under N_2_ atmosphere in a differential scanning calorimeter (TA Instruments Q2000, New Castle, DE). The densities (ρ) of the polished disks were measured according to Archimedes principle with deionized water as an immersion liquid. Vickers hardness values were determined using a Vickers microindenter (Everone MH-3, Everone Enterprises, Ltd., China) with a charge of 100 g for 5 s. Optical transmission spectra ranging from 0.5 μm to 16 μm were recorded using a PerkinElmer Lambda 950 spectrophotometer and a Nicolet 380 FT-IR spectrometer. Raman spectra were obtained at room temperature by using the back-scattering configuration of a laser confocal Raman spectrometer (type: Renishaw inVia, Gloucestershine, UK) with an excitation wavelength at 785 nm. The resolution of the Raman spectra was 1 cm^−1^. The as-prepared disks were sputtered with gold on both sides as electrodes, and the diameter of the electrodes was 8 mm. The ionic conductivity of the samples was evaluated with a Solarton 1260 frequency analyzer at 30 °C–150 °C, 1 Hz–1 MHz frequency, and 10 mV applied voltage amplitude.
